# Bibliometric analysis of vitamin D and obesity research over the period 2000 to 2023

**DOI:** 10.3389/fphar.2024.1445061

**Published:** 2024-07-18

**Authors:** Xudong Song, Senhua Qin, Shuxin Chen, Can Zhang, Lin Lin, Ziyi Song

**Affiliations:** ^1^ Guangxi Key Laboratory of Animal Breeding, Disease Control and Prevention, College of Animal Science and Technology, Guangxi University, Nanning, China; ^2^ Department of Gynecology, The Reproductive Hospital of Guangxi Zhuang Autonomous Region, Nanning, China

**Keywords:** vitamin D, obesity, vitamin D deficiency, trends, bibliometric analysis

## Abstract

**Background:**

Globally, the incidence rates of obesity and its related diseases, such as cardiovascular diseases and type 2 diabetes, are continuously rising, posing a significant public health challenge. Studies have indicated a potential correlation between vitamin D deficiency and obesity. However, a quantitative analysis of the studies related vitamin D and obesity is lacking. This investigation aims to fill this gap by providing a comprehensive bibliometric analysis to uncover the collaborative networks, research hotspots, and evolutionary trends within the field of vitamin D and obesity research.

**Methods:**

This study retrieved literature related to vitamin D and obesity from the Web of Science database spanning from 2000 to 2023. Bibliometric analysis was conducted using tools such as HistCite, VOSviewer, and CiteSpace to excavate multi-dimensional information including countries, institutions, authors, journals, citations, and keywords.

**Results:**

A total of 6,144 records were retrieved, involving 123 countries, 6,726 institutions, and 28,156 authors, published in 1,551 journals. The number of published papers and citations showed a generally increasing trend. The United States led in terms of publication volume and influence, with journals such as Nutrients and Obesity Surgery having the highest publication counts. Nasser M. Al-Daghri was the most prolific and influential author. Keyword clustering revealed that research topics covered metabolic health, nutrition, immunity, and bariatric surgery. Citation burst analysis indicated a shift in research focus from the relationship between dietary calcium and obesity to the preventive effects of vitamin D supplementation on metabolic diseases.

**Conclusion:**

The application of bibliometric methods to analyze the research literature in the fields of obesity and vitamin D has provided a comprehensive understanding of the collaborative networks, key research focus, and evolutionary trends in this field, offering insights for guiding future research directions.

## 1 Introduction

Obesity has emerged as a pressing global health concern, affecting diverse age groups and populations. It contributes to chronic conditions such as cardiovascular diseases, type 2 diabetes, and metabolic syndrome (Piché et al., 2020). According to a recent study published in The Lancet, by 2022, more than one billion people in the world are now living with obesity. Since 1990, the prevalence of obesity among adults worldwide has more than doubled, while the rate among children and adolescents (aged 5–19) has quadrupled (Phelps et al., 2024). Obesity has emerged as a major public health concern worldwide, necessitating the implementation of effective preventive and control measures to mitigate its impact on population health. Hence, it is vital to establish extensive research and multifaceted treatment approaches for obesity.

Vitamin D, a fat-soluble vitamin, exists in two primary forms: vitamin D_2_ (VD2) and vitamin D_3_ (VD3). Human vitamin D primarily originates from skin synthesis (VD3) and dietary intake (VD2 or VD3). To exert biological activity, vitamin D undergoes hydroxylation in the liver to form 25-hydroxyvitamin D (25(OH)D, the circulating form), followed by further hydroxylation in the kidneys to produce 1,25-dihydroxyvitamin D (1,25(OH)_2_D, the active form) (Kulda, 2012). It plays crucial roles in calcium-phosphate metabolism, immune modulation, cellular growth, differentiation, and apoptosis (Zmijewski, 2019). Additionally, emerging evidence suggests a potential anti-obesity role for vitamin D (Abdullah Thani et al., 2019). Vitamin D deficiency is more prevalent among obese individuals, and its role in the association between obesity and cancer risk has been suggested (Sánchez-Bayona et al., 2022). Evidence indicates that vitamin D may participate in the onset and progression of obesity by influencing fat metabolism, modulating hormone levels, and regulating inflammation and immune responses (Argano et al., 2023). Consequently, understanding the complex relationship between obesity and vitamin D has become a key area of research.

Bibliometric analysis is a method that quantifies and visualizes published literature. It involves the analysis of the quantity and quality of literature, publications, and citation information (Agarwal et al., 2016). This method aims to review the development, impact, and trends within scientific research. Combining approaches from informatics, statistics, sociology, and other disciplines, it seeks to quantify and evaluate the impact of research outcomes, authors’ contributions, and the dynamic changes within academic domains. Its applications include investigating the dynamics of literature production, assessing journal influences, determining citation patterns, and identifying research themes or future directions, including hot topics (Nicolaisen, 2010). In recent years, the utilization of bibliometric analysis as a scientific research tool has steadily increased in publication volume (Ellegaard and Wallin, 2015). For newcomers in a specific research field, timely and comprehensive systematic reviews offer valuable overviews of knowledge domains and guide effective initiation of research. For experienced and active researchers, systematic reviews aid in keeping abreast of the latest advancements in their field (Chen and Song, 2019). Scholarly analysis using bibliometric methods has explored the role of vitamin D in immunity (Luo et al., 2022), bone metabolism (Malik et al., 2022), infections (He et al., 2022), reproductive health (Lu et al., 2022), and non-alcoholic fatty liver disease (Wang and Chang, 2023). However, the relationship between vitamin D and obesity remains unexplored, which is particularly intriguing given the high prevalence of vitamin D deficiency in obese individuals and its potential implications for metabolic health. This study aims to bridge this gap by conducting a comprehensive bibliometric analysis of literature pertaining to vitamin D and obesity from 2000 to 2022. Utilizing advanced data visualization techniques, we will quantify and map the development and focus of this interdisciplinary research field, shedding light on the current research status and trends. Our findings are expected to not only elucidate the complex interplay between vitamin D and obesity but also provide a solid foundation and valuable insights for guiding future research directions and clinical applications.

## 2 Materials and methods

### 2.1 Search strategy in web of science core collection

**FIGURE 1:**
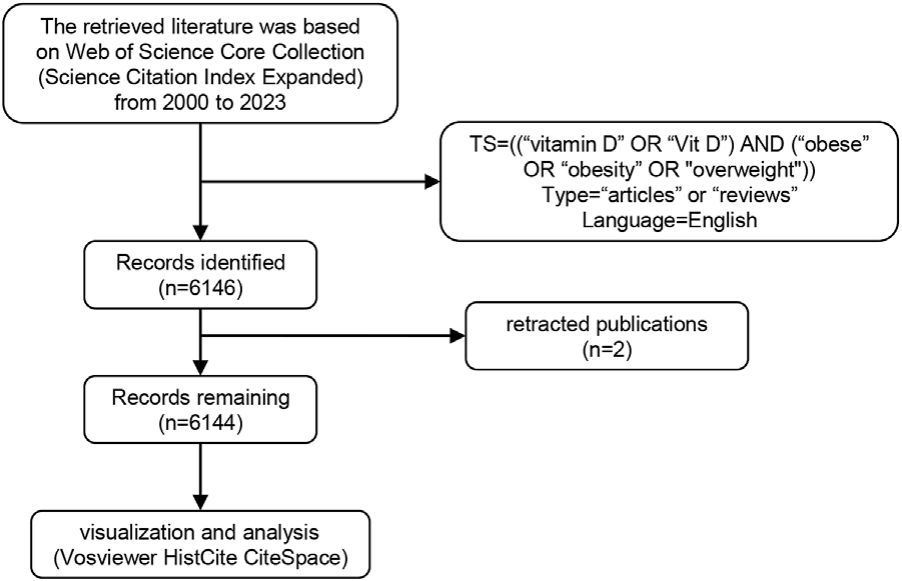
Flow chart of document selection and identification.

In this study, we have chosen the Web of Science Core Collection (WoSCC) as our data source. The Web of Science is regarded as the world’s largest and most comprehensive collection of information resources, and its standardized data structure and rich citation information make it particularly suitable for bibliometric analysis (Wang et al., 2020). To ensure the precision and relevance of our analysis, we have decided to focus exclusively on English-language articles from the WoSCC. Considering the various English expressions for “vitamin D” and “obesity” or “overweight”, we referenced keywords used by relevant researchers to effectively extract literature related to vitamin D and obesity or overweight. Our aim was to accurately encompass research articles on these topics within the WoSCC database. After multiple searches and comparisons, we finalized our search formula as “TS = ((“vitamin D” OR “Vit D”) AND (“obese” OR “obesity” OR “overweight”))”. We restricted publication years from 2000 to 2023, and the document types were “articles” or “reviews”. The article language was set as English. Search results were downloaded as “Full Record and Cited References” and “Plain Text”. The exported content was saved in a txt file for subsequent analysis. Initially, we obtained 6,146 publications, subsequently removing two retracted publications (see Supplementary Table S1). To avoid the potential bias caused by the continuous updating of the database, the search and export of files were all conducted within a single day (5 January 2024) (Figure 1).

### 2.2 Bibliometric analysis

HistCite (version 12.03.17) is a pivotal tool in bibliometric analysis, specializing in illustrating citation relationships among scholarly works (Garfield et al., 2006). Its functionalities encompass citation network graphs, timeline analyses, and in-depth citation metrics, offering insights into publication impact and research trends within academic domains. We imported the downloaded txt file into HistCite for analysis, yielding data on the quantity of publications per year, active countries, institutions, authors, and core publications, as well as the global total citation scores (TGCS) and local total citation scores (TLCS) for all results. TGCS represents citation counts within the Web of Science, while TLCS indicates citations concentrated within the current publication set. The results were summarized and organized using Excel 2021 (Microsoft). We also used Excel 2021 to visualize the trends in publication volume and the distribution of publications by country, providing a clear and interpretable representation of our results.

VOSviewer (version 1.6.19) is a specialized knowledge mapping tool used extensively in bibliometric analysis (Van Eck and Waltman, 2010). It is particularly adept at creating visual representations, such as network maps, time overlay maps, and density visualization maps, to reveal relationships among scholarly publications. Employing clustering analysis and co-occurrence networks, VOSviewer swiftly displays topic distributions, keyword associations, and collaboration networks, offering a comprehensive understanding of the scientific research landscape (Qiu et al., 2014). This paper primarily utilizes VOSviewer to visualize the countries, institutions, keywords, and authors of articles. To obtain the corresponding views, the literature data is imported into VOSviewer, and the content for visualization is selected. In cases where clustering is not apparent, Pajek (http://mrvar.fdv.uni-lj.si/pajek/) is used for adjustment. Additionally, Scimago Graphica (version 1.0.41) (Hassan-Montero et al., 2022) is employed to depict the publication volume of countries and international cooperation among states.

Citespace (version 6.2.R4) is a robust bibliometric analysis tool primarily focused on visualizing citation networks among scholarly publications, emphasizing the temporal and spatial relationships between works (Chen, 2006). Its key functionalities include identifying critical paths, significant nodes, and literature clustering, aiding in comprehending knowledge structures and research evolution (Wang et al., 2022). In the analysis of the field concerning vitamin D and obesity, CiteSpace primarily conducted burst analysis for references and keywords. This analytical approach helps to uncover research trends, emerging topics, and key turning points in the field, providing valuable insights for researchers and guiding future research directions. The time slice was set from January 2000 to December 2023, with a 2-year interval per slice. Node types were defined as “references” or “keywords”, selecting the top 50 levels based on the most cited or frequently occurring criteria within each time slice. Modularized Q and average silhouette were used to assess clustering reliability; where Q > 0.3 and average silhouette >0.5 indicate sufficient clustering structure and convincing clustering results. The criteria selected the top 50 levels based on the most cited or frequently occurring references and keywords within each time slice.

## 3 Result

### 3.1 The global growth trend of publication outputs in vitamin D and obesity research

**FIGURE 2:**
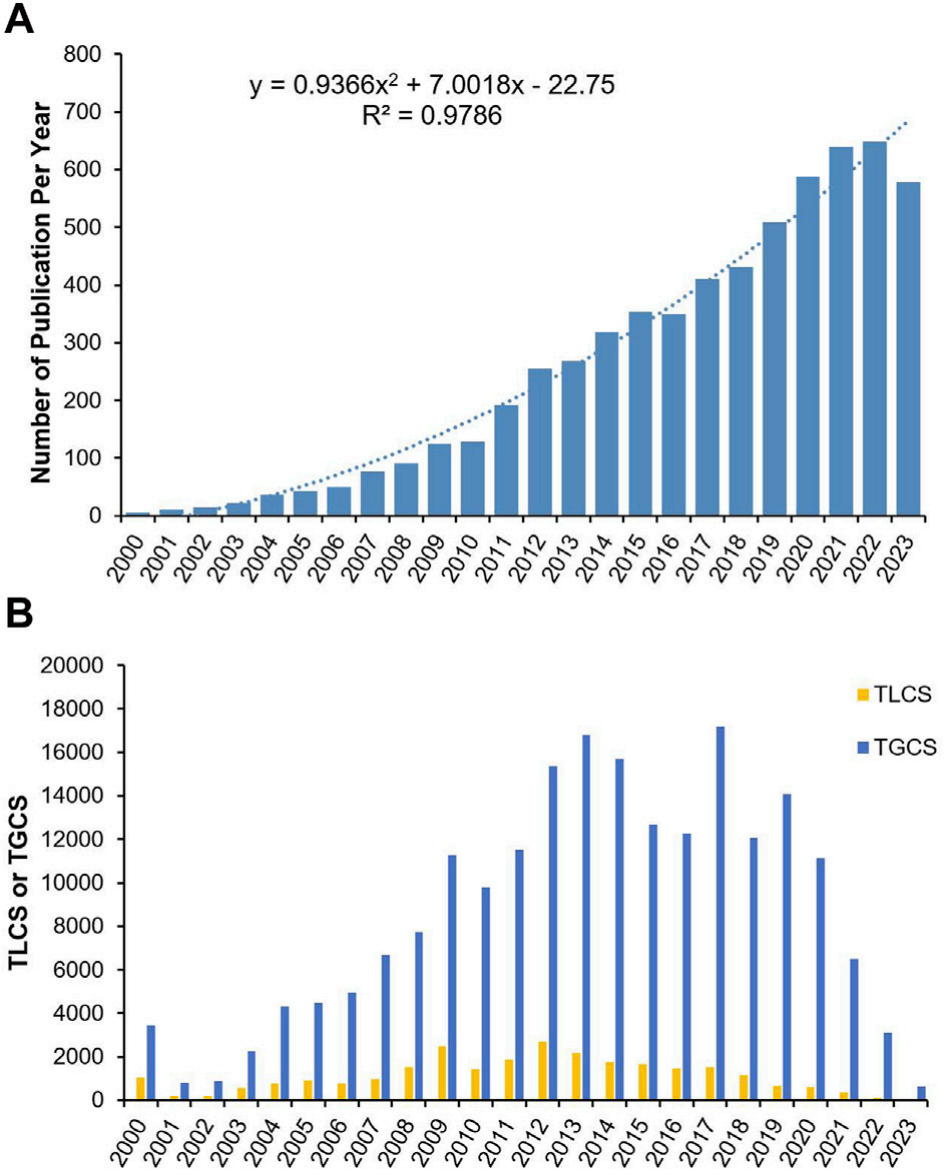
Publication outputs and citations on vitamin D and obesity in the past 23 years. **(A)** Global annual production trends. Blue bars represent the number of publications per year. **(B)** Annual TGCS and TLCS of publications.

Between 2000 and 2023, a total of 6,144 publications concerning vitamin D and obesity were retrieved from WoSCC, after excluding two rejected articles. This collection comprises 4,881 original research articles (accounting for 79.44%) and 1,263 review articles (accounting for 20.56%). With the exception of minor fluctuations in 2016, there has been a consistent uptrend in publication numbers from 2000 to 2023, reaching a peak in 2022 with 642 publications. Notably, there was significant growth during the intervals of 2010 to 2012 and 2018 to 2021 (Figure 2A). In general, although the early stage of development featured a modest number of publications, the volume of citations was remarkably high. In the year 2000, a modest sum of six articles were published, of which the study conducted by Wortsman et al. (cited 2,258 times) uncovered a potential link between vitamin D and obesity (Wortsman et al., 2000), stimulating an increase in subsequent research on this topic. Corresponding to the increase of publications, the TGCS and TLCS have been relatively high since 2000. The TGCS demonstrated a progressive increase from 2001, reaching its peak in 2013. Despite notable variations in 2017, the TGCS and TLCS have been relatively stable since 2015, corroborating that research into vitamin D and obesity remains a popular topic to date (Figure 2B).

### 3.2 Analysis of country contribution and country burst in vitamin D and obesity research

**FIGURE 3:**
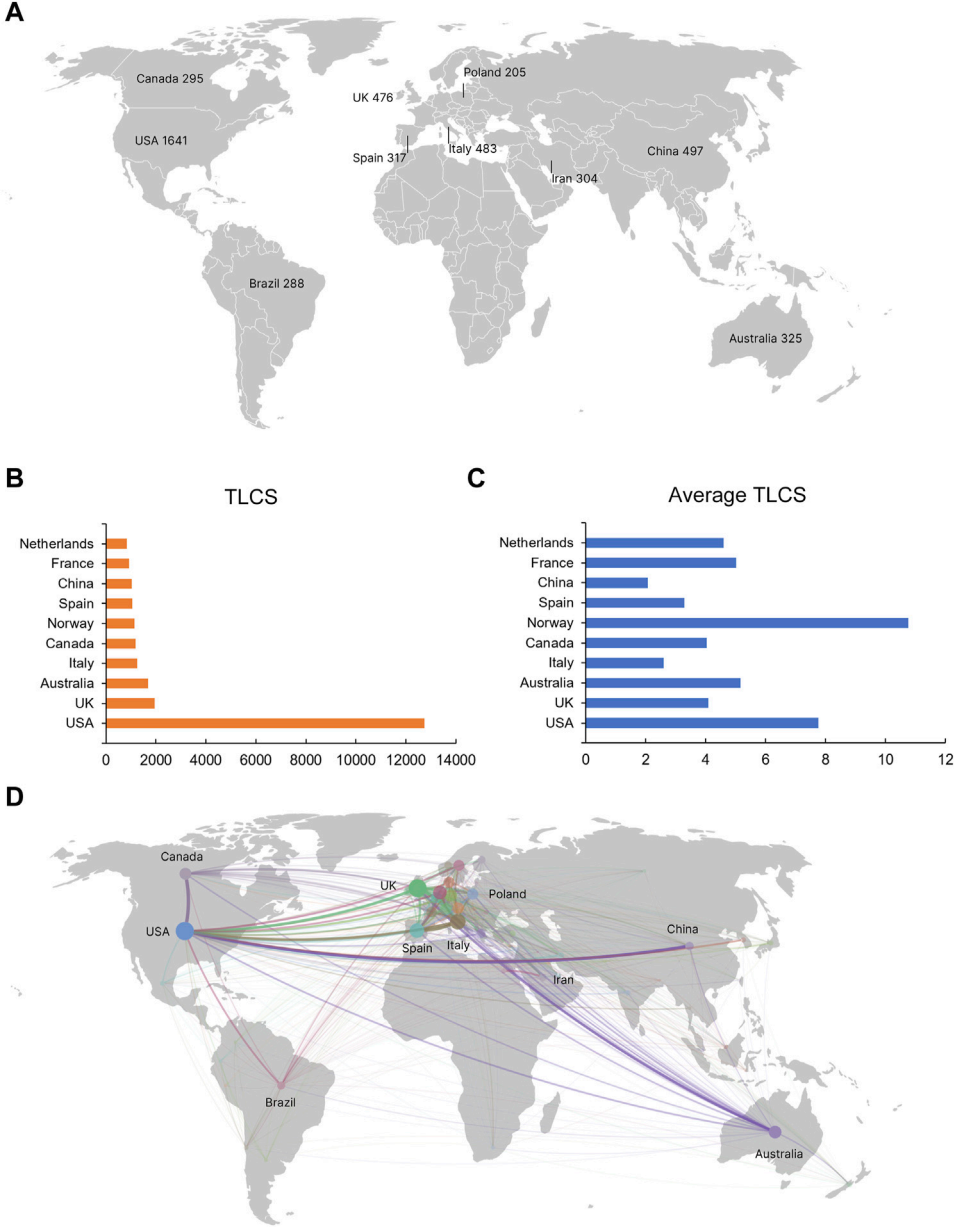
The leading country in the field of vitamin D and obesity research. **(A)** Top 10 countries by the number of publications. **(B)** Top ten countries with the highest TLCS. **(C)** Top ten countries with the highest average TLCS. **(D)** International cooperation among States. Each country is represented as a node, and each line represents a co-author relationship. The size of the node is proportional to the strength of the cooperative link.

It was found that 123 countries and regions participated in the study of vitamin D and obesity. The five countries that contributed the most publications were the United States (1,641), China (497), Italy (483), the United Kingdom (476), and Australia (325) (Figure 3A) (Table 1). In the TLCS ranking, the United States still ranks first (12,734 citations), followed by the United Kingdom (1,946), followed by Australia (1,675), Italy (1,256) and Canada (1,190) (Figure 3B). Norway, notwithstanding not having the highest publication or TLCS volumes, stood out with the highest average TLCS (Figure 3C), indicating its considerable impact. There were 65 countries with more than ten publications that were included in the co-authorship analysis. The highest total link strength was observed in the United States (total link strength = 866 times) (Figure 3D), In this largest cooperative network led by the United States, the United Kingdom (838), Italy (626), Spain (551), and Netherlands (481) were in key positions. Despite China’s considerable contribution to the volume of publications, the nation manifests a total link strength of merely 184, reflecting a comparatively restrained impact within the scope of global research collaborations.

**TABLE 1:** Analysis of top ten countries with the highest TLCS.

Rank	Country	Publications	TLCS	TGCS	Average TLCS
1	United States	1,641	12,734	90,369	7.76
2	United Kingdom	476	1,946	23,660	4.09
3	Australia	325	1,675	12,851	5.15
4	Italy	483	1,256	15,239	2.60
5	Canada	295	1,190	11,581	4.03
6	Norway	105	1,131	5,261	10.77
7	Spain	317	1,041	11,809	3.28
8	China	497	1,029	9,448	2.07
9	France	184	923	11,939	5.02
10	Netherlands	182	838	12,590	4.60

### 3.3 Active institutes in vitamin D and obesity research

**FIGURE 4:**
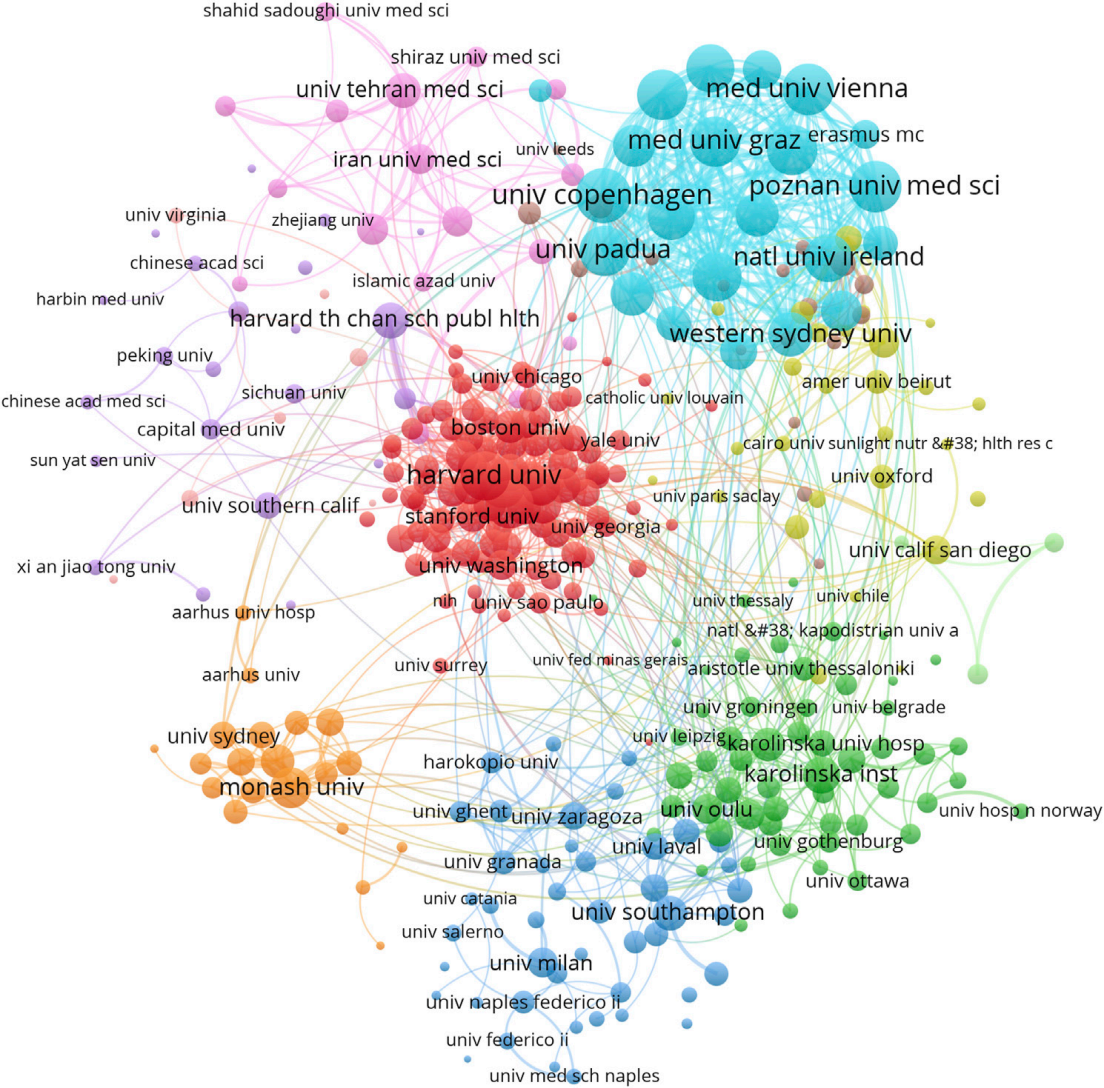
Active institutional analysis. Each organization represents a node, the size of the node is proportional to the strength of the cooperative link, each line represents a co-authoring relationship, and the line thickness indicates the strength of the collaborative link.

A total of 6,726 institutions contributed to publications in this field. The five institutions of the most contributed publications are: Harvard University (104 publications), University of Tehran Medical Sciences (96), King Saud University (84), Harvard Medical School (78), and Monash University (75) (Table 2). The top 10 productive institutions are mainly distributed in the United States (3 institutions) and Iran (2 institutions). Strikingly, the United States has a staggering 9 of the top 10 institutions in TLCS (Table 2). We further screened 334 institutions with more than 10 papers, excluding 1 institution with no relationship, and the institutional collaboration network is mainly divided into 11 main clusters (Figure 4). The top institutions are located in the upper right cluster, and the University of Copenhagen had the most cooperation with other institutions (total link strength = 304), followed by Medical University of Vienna (260), Poznan University of Medical Sciences (255), University of Padua (249), and Medical University of Graz (242).

**TABLE 2:** Analysis of top ten Institution with the highest TLCS.

Rank	Institution	Publications	TLCS	TGCS	Average TLCS
1	Boston University	44	1,643	4,800	37.34
2	Harvard University	104	1,353	10,438	13.01
3	Tufts University	45	653	3,244	14.51
4	University of Tennessee	46	645	3,237	14.02
5	University of Tromso	20	506	1,906	25.3
6	University of Minnesota	33	493	1,666	14.94
7	Creighton University	23	482	2,175	20.96
8	Mayo Clinic	54	449	2,449	8.31
9	Brigham and Women’s Hospital	71	432	5,310	6.08
10	University of California, Los Angeles	24	420	2,919	17.5

### 3.4 Active authors in vitamin D and obesity research

**FIGURE 5:**
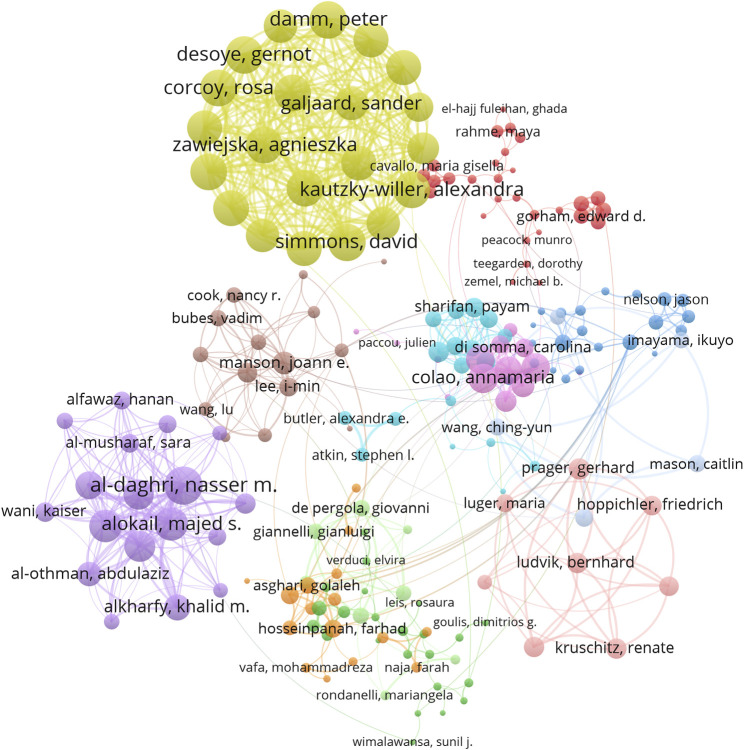
Analysis of active author. Each author represents a node, the size of the node is proportional to the strength of the collaboration link, each line represents a co-authoring relationship, and the line thickness indicates the strength of the collaboration link. The color of a node represents the cluster it belongs to.

A total of 28,156 authors contributed to publications in this field, with Nasser M. Al-Daghri from King Saud University being the most prolific, having published 44 papers in this field (Table 3). The author with the highest LTCS was Michael F. Holick (1,288) from Boston University (Supplementary Table S2). We further conducted co-authorship analysis on 433 authors with more than 5 publications, resulting in 197 authors after excluding 52 irrelevant authors. These authors were then divided into 13 largest collaborative networks. The highest total link strength was observed for Nasser M. Al-Daghri with a total link strength of 172, followed by Gernot Desoye (163), Peter Damm (162), David Simmons (162), and Alexandra Kautzky-Willer (160) (Figure 5). These authors are leading authorities in the collaborative research domain of vitamin D and obesity.

**TABLE 3:** Analysis of top 10 productive authors.

Rank	Name	Institutions	Publications	TLCS	TGCS
1	Nasser M. Al-Daghri	King Saud University	44	45	1,140
2	Giovanna Muscogiuri	The Catholic University of America	29	251	1,133
3	Yue Chen	Hunan Normal University	28	296	883
4	Wang Y	Johns Hopkins University	28	76	436
5	Colao A	Università degli Studi di Napoli Federico II	26	112	830
6	Alokail MS	King Saud University	25	1	719
7	Luigi Barrea	Università degli Studi di Napoli Federico II	24	111	729
8	Y Zhang	Xi’an Jiaotong University	24	25	457
9	Omar S Al-Attas	King Saud University	22	1	654
10	Barbora de Courten	Monash University	22	155	664

### 3.5 Core journals in vitamin D and obesity research

**FIGURE 6:**
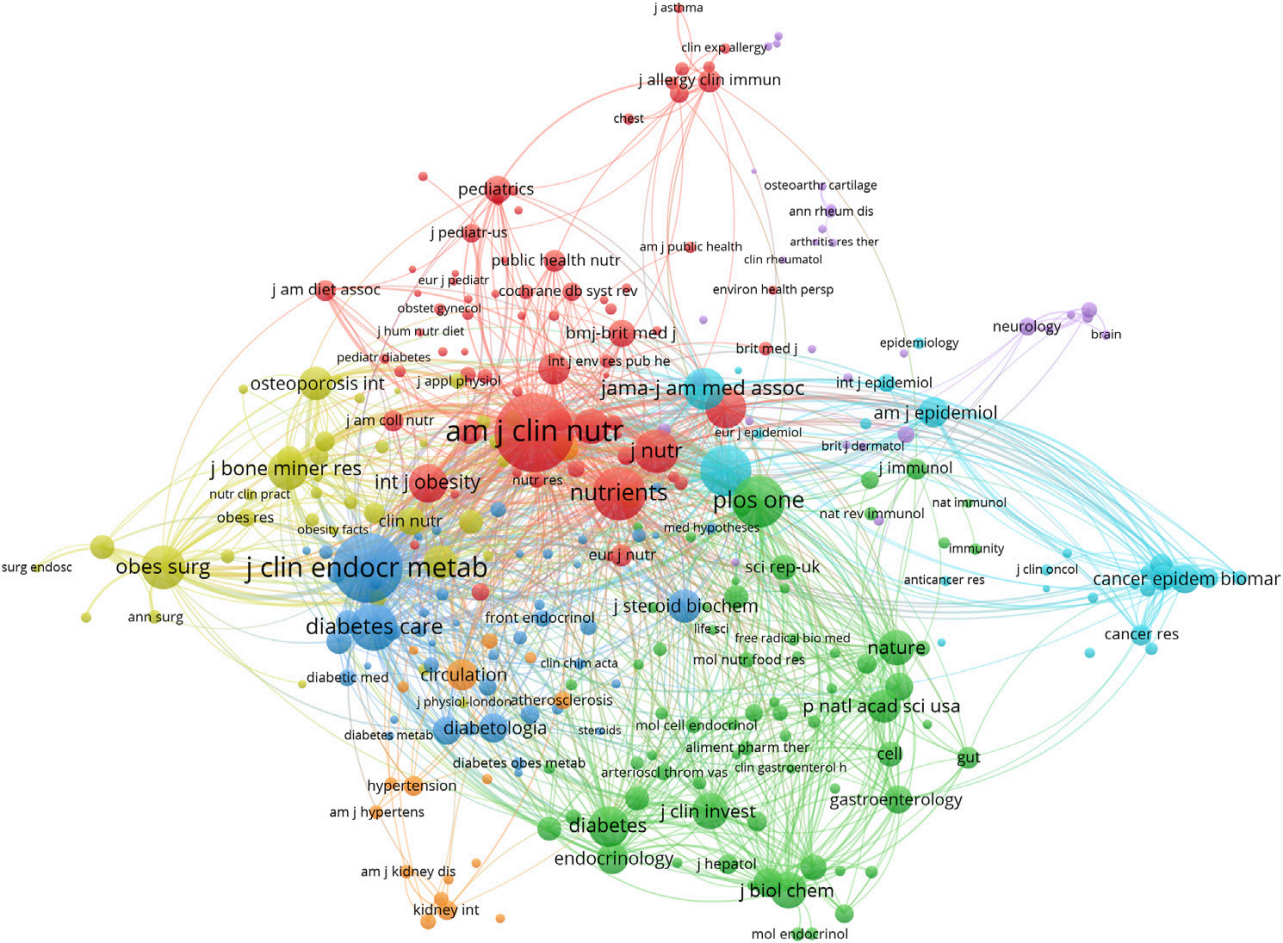
Analysis of core journals. Each node represents a journal and each row represents a co-citation relationship. The node size is proportional to the strength of the co-referenced link, and the node color reflects the cluster it belongs to.

Studies on the relationship between vitamin D and obesity have been published in 1,551 journals, and the five journals with the largest number of literature in this field are Nutrients (382 articles), Obesity Surgery (204), PLOS ONE (99), Journal of Clinical Endocrinology and Metabolism (82), and American Journal of Clinical Nutrition (70) (Table 4). These publications account for approximately 20.64% of all the papers on this topic. Although PLOS ONE, Frontiers in Endocrinology, and International Journal of Molecular Sciences have published a large number of articles, these articles have a relatively low TLCS in the local literature on vitamin D and obesity. It is worth noting that although American Journal of Clinical Nutrition (67 articles) ranks only fifth in the number of articles contributed, it is the highest journal in TLCS, with 2,436 TLCS, followed by Obesity Surgery (2,050) and the Journal of Clinical Endocrinology and Metabolism (2,004) (Supplementary Table S3). The top ten journals with the highest TLCS account for approximately 38.18% of the overall TLCS, underscoring their significant influence and demonstrating their pivotal role in the research of vitamin D and obesity. The co-citation analysis encompassed 299 journals each cited over 200 times. The American Journal of Clinical Nutrition (Total Link Strength = 663,815), The Journal of Clinical Endocrinology and Metabolism (540,670), Nutrients (337,032), PLOS ONE (320,538), and The New England Journal of Medicine (308,467) were the most frequently co-cited with other journals (Figure 6).

**TABLE 4:** Analysis of top 10 productive journal.

Rank	Journal	Recs	TLCS	TGCS	Impact factor (2022–2023)	H Index
1	Nutrients	382	239	7,435	5.9	75
2	Obesity Surgery	204	2,050	7,036	2.9	128
3	PLOS ONE	99	0	3,635	3.7	268
4	Journal of Clinical Endocrinology and Metabolism	82	2,004	6,903	5.8	328
5	American Journal of Clinical Nutrition	70	2,436	8,948	7.1	307
6	British Journal of Nutrition	62	398	2,280	3.6	166
7	Frontiers in Endocrinology	61	0	682	5.2	51
8	International Journal of Molecular Sciences	60	21	1,370	5.6	114
9	Journal of Steroid Biochemistry and Molecular Biology	57	507	1,512	4.1	116
10	European Journal of Clinical Nutrition	56	321	1,774	4.7	141

### 3.6 Keywords analysis in vitamin D and obesity research

**FIGURE 7:**
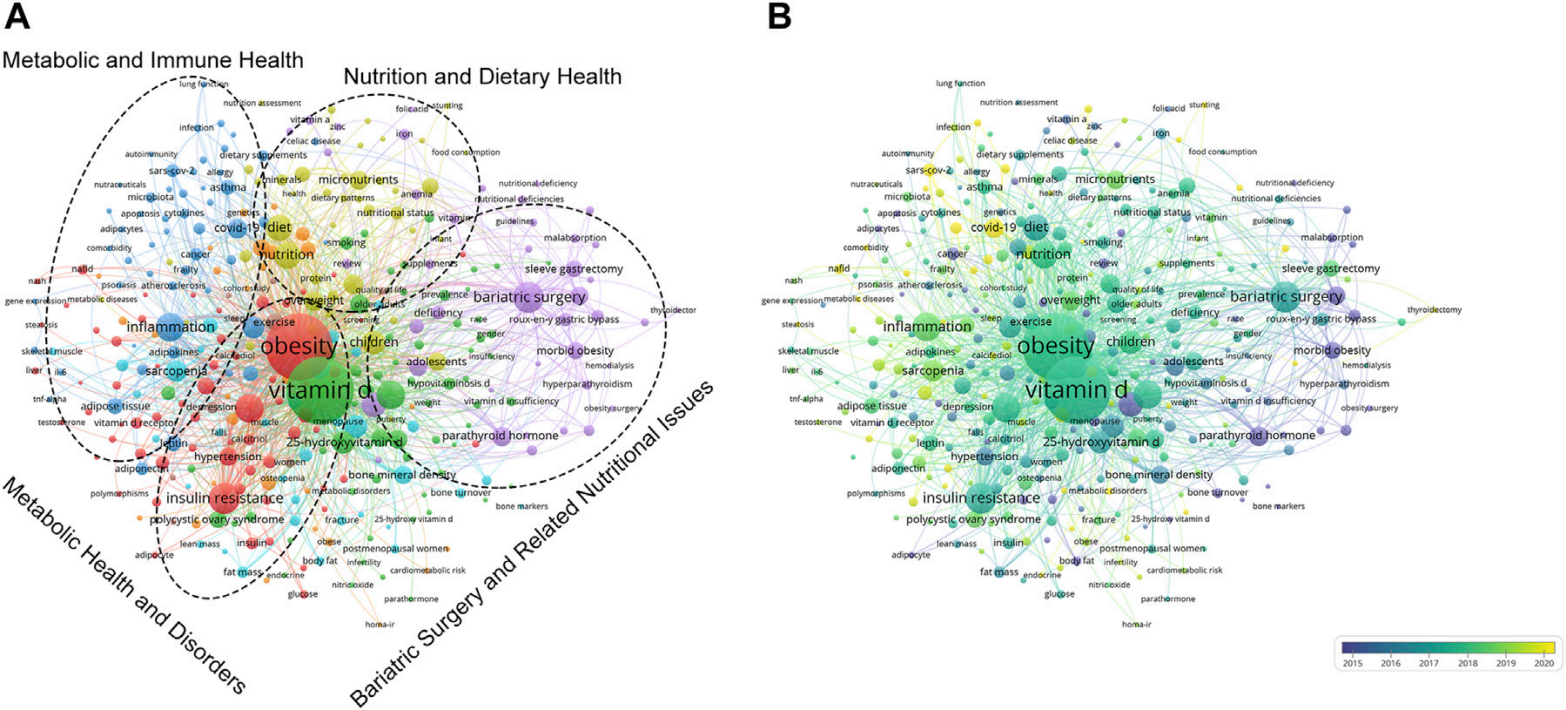
Visualization map of keywords. **(A)** Divide keywords into four different clusters by topic. **(B)** Dynamics and trends of the keywords.

**FIGURE 8:**
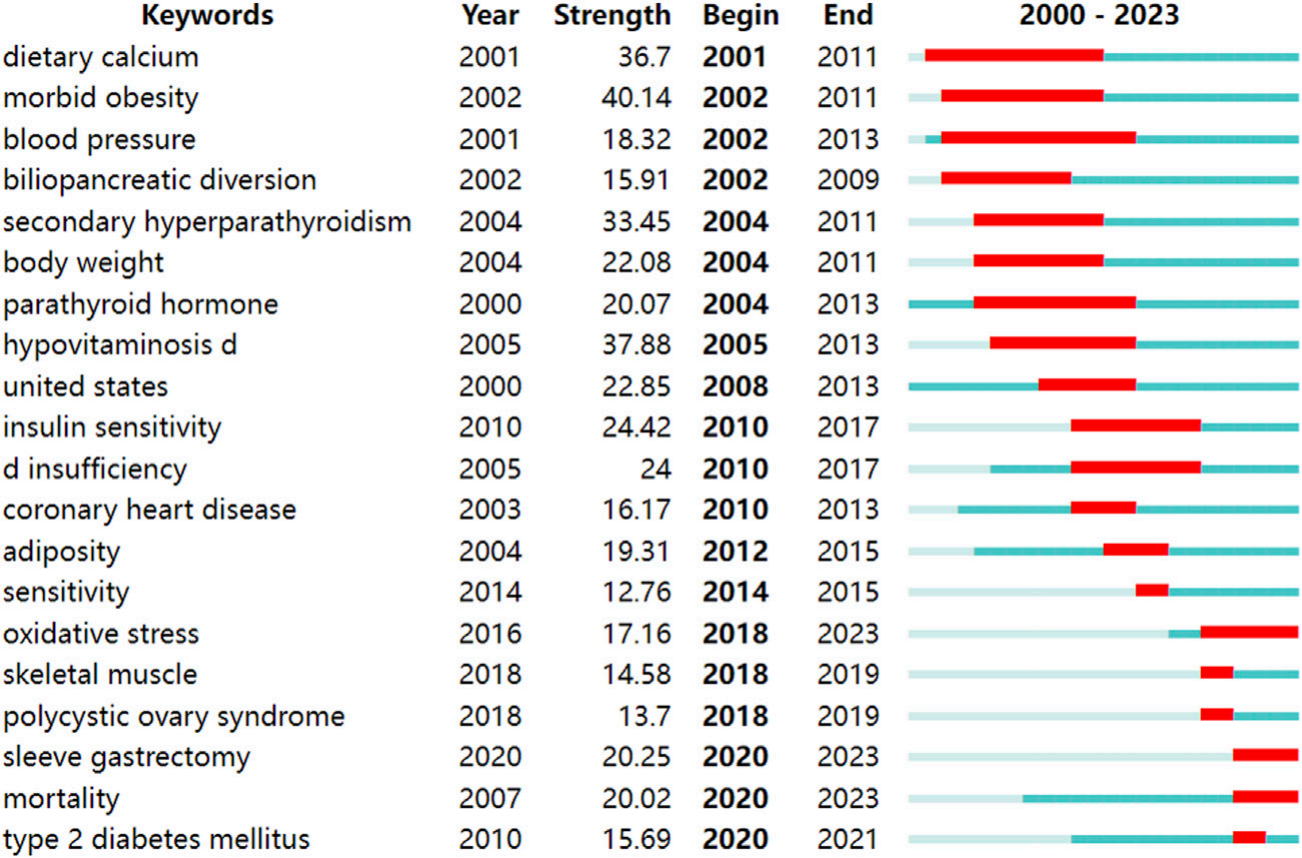
Top 20 keywords with the strongest citation bursts.

A total of 341 keywords (set as author keywords) were identified as having occurred more than ten times. According to the clustering, the research topics in vitamin D and obesity can be roughly divided into four parts: metabolic health and disorders, nutrition and dietary health, metabolic and immune health, and bariatric surgery and related nutritional issues (Figure 7A). Additionally, on the timeline view, it was found that “stunting”, “COVID-19”, and “biomarker” are recent research trends (Figure 7B). We also conducted keyword burst detection and extracted 68 keywords with high burst intensity. The top 20 keywords are shown in Figure 8. The evolution of research topics in the field encompassing vitamin D and obesity is outlined by citation bursts, illustrating a shift in focus over the years. In the period from 2000 to 2010, initial studies focused on the association between dietary calcium and morbid obesity, and began to investigate the relationship between blood pressure and obesity. Additionally, the surgical approach of biliopancreatic diversion was explored as a treatment for severe obesity. There was also an increasing focus on issues of obesity related to secondary hyperparathyroidism, as well as studies centered on body weight, parathyroid hormone, and hypovitaminosis D. In the years spanning 2010 to 2017, research focus shifted to the impact of insulin sensitivity and vitamin D insufficiency on the risk of coronary heart disease, and the study of the relationship between vitamin D insufficiency and obesity. Molecular insights gained prominence in the years 2018–2019, as the direction of research gravitated more towards the cellular and molecular levels with the inclusion of oxidative stress, skeletal muscle, and polycystic ovary syndrome, illustrating a shift from macro-level studies to exploring underlying micro-mechanisms. In the current stage from 2020 to 2023, novel therapeutic and pathophysiological research methods emerged, such as the adoption of sleeve gastrectomy in weight reduction surgeries. Simultaneously, the long-term effects of mortality and diabetes mellitus continued to be scrutinized. The research field of vitamin D and obesity has transitioned from early dietary concerns related to obesity to in-depth explorations of treatment strategies, cellular mechanisms, and the long-term impacts on health.

### 3.7 Analysis of highly cited articles in vitamin D and obesity research

The top 20 publications with the highest TLCS are presented in Table 5. In addition to two reviews, the rest are research articles, most of which focus on the study of obesity and vitamin D levels in the body. Among of them, seven articles are related to the relationship between obesity and vitamin D levels, four articles are about the treatment of obesity with vitamin D supplement, four articles are about the role of vitamin D in adipose tissue, and three articles are about vitamin D receptor (VDR) research. The most cited article is the one published by Jacobo Wortsman et al. in the American Journal of Clinical Nutrition (Wortsman et al., 2000), which found that the bioavailability of vitamin D_3_ in obese individuals is reduced, possibly due to its deposition in adipose tissue, leading to a higher prevalence of vitamin D deficiency in obese individuals. The article also suggests that the vitamin D supplement dose for obese individuals may need to be larger (Wortsman et al., 2000). This article has laid the foundation for the study of vitamin D and obesity, and serves as a pioneer in this research field.

**TABLE 5:** Top 20 publications with the highest TLCS.

Rank	First author	Journal	Year	TLCS	TGCS
1	J Wortsman	The American Journal of Clinical Nutrition	2000	1,042	2,258
2	Shamik J Parikh	The Journal of Clinical Endocrinology and Metabolism	2004	266	479
3	Andjela T Drincic	Obesity	2012	256	426
4	Marieke B Snijder	The Journal of Clinical Endocrinology and Metabolism	2005	231	523
5	Sonia Arunabh	The Journal of Clinical Endocrinology and Metabolism	2003	228	470
6	Susan Cheng	Diabetes	2010	204	370
7	C P Earthman	The International Journal of Obesity	2012	195	305
8	Ramin Alemzadeh	Clinical and Experimental	2008	167	317
9	Armin Zittermann	The American Journal of Clinical Nutrition	2009	141	412
10	Miriam Blum	International Journal of Endocrinology and Metabolism	2008	139	296
11	Simon Vanlint	Nutrients	2013	134	250
12	Cherlyn Ding	The British Journal of Nutrition	2012	131	226
13	M F McCarty	Medical Hypotheses	2003	123	216
14	L Wamberg	The International Journal of Obesity	2013	115	166
15	Jared P Reis	Pediatrics	2009	114	267
16	Christy B Turer	Pediatrics	2013	114	198
17	Anthony M Belenchia	The American Journal of Clinical Nutrition	2013	109	210
18	Kari E Wong	Endocrinology and Metabolism	2009	105	194
19	Kari E Wong	The Journal of Biological Chemistry	2011	103	140
20	Carmen J Narvaez	Endocrinology	2009	102	177

### 3.8 Analysis of publications in vitamin D and obesity research

**FIGURE 9:**
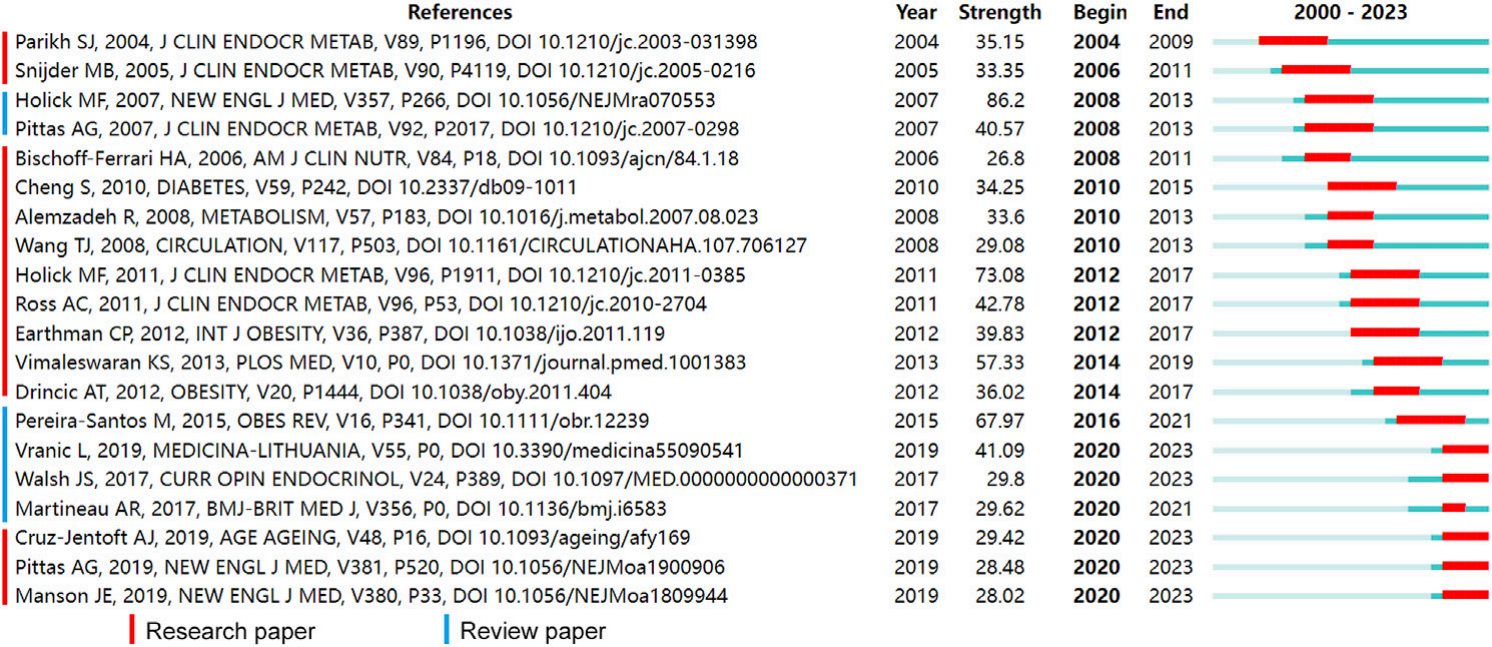
Top 20 references with the strongest citation bursts.

We conducted a citation burst detection from 2000 to 2023, identifying the top 20 references with the strongest citation bursts as shown in Figure 9. Most of the publications are research articles, accompanied by 5 reviews. From 2004 to 2009, influential articles by Parikh et al., Snijder et al., and Alemzadeh et al. predominantly discussed the association between vitamin D levels and obesity (Parikh et al., 2004; Snijder et al., 2005; Alemzadeh et al., 2008), as well as the impact of dietary calcium supplementation on obesity. Building upon these studies, work by Wang, T. J. et al. and Cheng et al. suggested links between vitamin D and cardiovascular diseases as well as metabolic disorders (Wang et al., 2008; Cheng et al., 2010). Besides, investigations by Bischoff-Ferrari et al. and Ross et al. provided insights on optimal serum levels of vitamin D and recommended dietary intakes (Bischoff-Ferrari et al., 2006; Ross et al., 2011). Between 2010 and 2016, the causal relationship between obesity and vitamin D deficiency was further investigated by Drincic et al., Pereira-Santos et al., and Vimaleswaran et al. (Drincic et al., 2012; Vimaleswaran et al., 2013; Pereira-Santos et al., 2015). From 2017 to 2020, Cruz-Jentoft et al. discussed the significance of vitamin D among the elderly and patients with sarcopenia (Cruz-Jentoft et al., 2019). Research by Manson et al. and Pittas et al. explored the potential roles of vitamin D supplementation in the prevention of cancer and type 2 diabetes, respectively (Manson et al., 2019; Pittas et al., 2019).

### 3.9 The evolving trends of research on vitamin D and obesity

In 2000, Wortsman et al. uncovered lower baseline 25(OH)D levels in obese individuals, hinting at a possible link between obesity and vitamin D (Wortsman et al., 2000). Subsequently, in 2001, Shi et al. reported the impact of 1,25(OH)_2_D on calcium ion signaling in adipocytes (Shi et al., 2001), which governs their lipogenesis and lipolysis. Furthermore, the nongenomic pathway mediated by VDR may represent a crucial target for the development of obesity treatment interventions. However, the precise mechanisms remained elusive for years, sparking a surge in interest in understanding the relationship between vitamin D and obesity, leading to an expanding body of research. Findings from Arunabh et al., Parikh et al., and Snijder et al. further supported the negative correlation between vitamin D levels and body weight across different demographic groups (Arunabh et al., 2003; Parikh et al., 2004; Snijder et al., 2005). In 2006, Hyppönen and Power suggested that vitamin D status might impact glucose metabolism, potentially contingent on body size, paving new paths for investigation (Hyppönen and Power, 2006). By 2008, Sneve et al. noted that supplementing cholecalciferol in individuals did not significantly reduce weight among overweight or obese subjects (Sneve et al., 2008). This study strongly suggested a limited association between vitamin D supplementation and weight reduction but hinted at a potential preventive role. In the same year, multiple studies unveiled a close link between vitamin D deficiency, abnormalities in glucose metabolism, and obesity, particularly in children and adolescents (Alemzadeh et al., 2008; Goldner et al., 2008). This raised awareness regarding vitamin D’s potential role in metabolic diseases. In 2009, several studies delved into VDR functionality within adipose tissue and its impact on energy metabolism and inflammation, shedding further light on the connection between vitamin D and obesity (Narvaez et al., 2009; Wong et al., 2009; Zittermann et al., 2009). From 2010 to 2013, researchers further explored the intricate relationship between vitamin D and obesity alongside related metabolic diseases (Cheng et al., 2010). This encompassed its effects on visceral obesity, inflammation, insulin resistance, and offered new perspectives on correcting vitamin D deficiency in the treatment of obesity and its associated metabolic abnormalities. Since 2014, vitamin D deficiency has been observed more frequently in obese individuals, suggesting a connection between vitamin D levels and obesity (Pereira-Santos et al., 2015; Walsh et al., 2016). However, the causal relationship remains unclear. Vitamin D supplementation appears to impact obesity-related factors such as blood pressure, glucose levels, and insulin resistance (Mousa et al., 2017). Mechanistically, vitamin D is implicated in regulating fat synthesis, adipocyte differentiation, and energy expenditure (Roth et al., 2018). Obese individuals often exhibit lower serum vitamin D levels, potentially due to factors like vitamin D storage in adipose tissue and impaired hepatic hydroxylation processes (Roizen et al., 2019). In summary, while vitamin D deficiency may contribute to obesity, and *vice versa*, the intricate mechanisms underlying this relationship warrant further investigation.

## 4 Discussion

This study conducted a bibliometric analysis to clarify the collaborative networks, research trends, and hot topics in the field of vitamin D and obesity. The findings revealed that this field of study has garnered extensive global attention, with a total of 6,144 records retrieved, involving 123 countries, 6,726 institutions, and 28,156 authors, published in 1,551 journals. From the research results, while the initial publications in the field of vitamin D and obesity were not substantial, a pivotal study published in 2000, titled “Decreased bioavailability of vitamin D in obesity” (Wortsman et al., 2000) established a potential correlation between vitamin D deficiency and obesity. This study became an early key literature in the field and laid the foundation for subsequent research. Over the past 23 years, the annual publication volume of related literature has continued to increase, reaching a peak of 648 articles in 2022, indicating the sustained attention and importance of this field. This trend suggests that vitamin D and obesity research holds enduring academic value and social impact, and it will continue to be an area of significant academic importance in the future.

In terms of national output, the United States, China, Italy, the United Kingdom, and Australia are the top five countries in terms of publication volume in the field of vitamin D and obesity research, indicating their significant research contribution in this area. Their leading position in publication volume highlights their important influence in this field. Although Norway does not have the highest number of publications or TLCS, its average TLCS value is among the highest, indicating that Norway’s research in this field has significant academic influence. The United Kingdom exhibits a high intensity in the research collaboration network and has close collaboration with the United States, showing its advantage in scientific collaboration. However, although China has a large number of publications, its total link strength in the global collaboration network is relatively low, primarily collaborating with the United States, while its collaboration links with other countries are relatively weak, indicating that China’s global collaborative influence in the field of vitamin D and obesity needs to be further enhanced.

The study also found that among the institutions with the highest publication volume in the field of vitamin D and obesity research, the United States accounts for three of them: Harvard University, Harvard Medical School, and Brigham and Women’s Hospital, indicating the significant research advantage of American institutions in this field. In addition, universities such as Copenhagen University, Medical University of Vienna, Medical University of Poznan, University of Padua, and Medical University of Graz have shown outstanding performance in research collaboration. Moreover, in the institutional collaboration network, research institutions in Europe and the United States have formed two significant clusters, indicating that American and European institutions are at the forefront of research in the field of vitamin D and obesity. Nasser M. Al-Daghri from King Saud University is the author with the highest number of publications in this field, reflecting his significant influence in this area. His research focuses mainly on the relationship between vitamin D deficiency and cardiometabolic health, as well as the connection between vitamin D and metabolic health, making significant contributions to these fields. Michael F. Holick from Boston University is the author with the highest TLCS, and “Decreased bioavailability of vitamin D in obesity” is his representative work, for which he is the corresponding author. The TLCS of the authors related to this article are all relatively high, which shows the influence of this article in this field.

Research on vitamin D and obesity is mainly published in journals such as “Nutrients”, “Obesity Surgery”, “PLOS ONE”, “Journal of Clinical Endocrinology and Metabolology”, and “The American Journal of Clinical Nutrition”. These journals cover a wide range of research directions including nutrition, endocrinology, epidemiology, and surgical treatment, providing a comprehensive perspective and in-depth analysis for the field of vitamin D and obesity. Among them, “The American Journal of Clinical Nutrition” holds a central position in this study with the highest collaboration relationships and total link strength, indicating its high professional reference value. It focuses mainly on research in the fields of nutrition and dietetics, publishing the latest studies on nutrition, nutrition and disease, and energy metabolism.

Through keyword cluster analysis, the research topics on vitamin D and obesity can be roughly divided into four parts: metabolic health and disorders, nutrition and dietary health, metabolic and immune health, and bariatric surgery and related nutritional issues, indicating the central role of metabolism and nutrition in vitamin D and obesity research. From the keyword bursts, it can be seen that early research focused on the association between “dietary calcium” and “morbid obesity”, as well as the physiological processes centered around “blood pressure” and “parathyroid hormone”. Subsequently, the research focus shifted to “insulin sensitivity”, with an increasing emphasis on cellular and molecular levels, covering areas such as “oxidative stress”, “skeletal muscle”, and “polycystic ovary syndrome”, as well as the adoption of “pathophysiological” research methods. This shift marks a transition from observing macroscopic phenomena to delving into underlying microscopic mechanisms. Meanwhile, “type 2 diabetes”, a common complication of obesity, has continued to receive widespread research attention.

According to citation bursts, early research on vitamin D primarily focused on the correlation between vitamin D levels and obesity, as well as the impact of dietary calcium supplementation on obesity. Over time, mid-term research began to delve deeper into the causal relationship between obesity and vitamin D deficiency, and studied the role of vitamin D in preventing acute respiratory infections and enhancing immune system function. In the later stages, the research scope expanded further, focusing on the importance of vitamin D in the elderly and patients with muscle wasting syndrome, as well as the potential role of vitamin D supplementation in preventing cancer and type 2 diabetes. Overall, vitamin D research has shifted from basic associative studies to more applied prevention and treatment strategies.

In summary, the field of vitamin D and obesity research continues to attract attention and has become a hot research area. The establishment of research collaboration networks, the enhancement of scientific research strength, and diverse research directions have provided a solid foundation for the development of this field. Future research should continue to deepen our understanding of the relationship between vitamin D and obesity, and explore effective prevention and intervention strategies.

## 5 Limitations

Our study has several limitations. Firstly, our focus on vitamin D and obesity means that our analysis is based on the Web of Science Core Collection and includes only English articles and reviews related to this topic, which may result in selection bias. Secondly, the results from VOSviewer and CiteSpace are based on machine algorithms, which may introduce algorithmic bias. Lastly, the assessment of research progress relies primarily on the HistCite tool, which may not have captured all potential advancements and trends, thus there is a certain degree of informational bias.

## 6 Conclusion

The regulatory role of vitamin D in adipocyte differentiation, fat storage, and metabolism is pivotal in understanding its connection to obesity. Vitamin D exerts multifaceted regulation within the human body, impacting intricate mechanisms such as insulin resistance, immune response, and inflammatory pathways, which are crucial in studying the relationship between vitamin D and obesity. The influence of vitamin D supplementation on obesity and its associated metabolic disorders continues to be a significant area of investigation within the scientific community. Employing bibliometric analysis to survey the scholarly literature can provide researchers with valuable insights into the collaborative networks, key research focal points, and emerging trends within this field of study.
