# Supplementary material for: Bibliometric analysis of vitamin D and obesity research over the period 2000 to 2023
Source: Front Pharmacol. 2024 Jul 18;15:1445061. doi: 10.3389/fphar.2024.1445061 (PMC11291317; doi:10.3389/fphar.2024.1445061)
Supplement: Supplementary file 1 [file Table1.DOCX]

Supplementary Material

**Bibliometric analysis of vitamin D and obesity research over the period 2000 to 2023**

Xudong Song^1†^, Senhua Qin^1†^, Shuxin Chen^1^, Can Zhang^1^, Lin Lin^2^, Ziyi Song^1^

^1^Guangxi Key Laboratory of Animal Breeding, Disease Control and Prevention, College of Animal Science and Technology, Guangxi University, Nanning 530004, China

^2^Department of Gynecology, The Reproductive Hospital of Guangxi Zhuang Autonomous Region, Nanning 530029, China

***Correspondence:**
Ziyi Song

[Ziyi.Song@gxu.edu.cn](mailto:Ziyi.Song@gxu.edu.cn)

Lin Lin

linlin19830422@163.com

**^†^**These authors have contributed equally to this work and share first authorship

**Supplementary Table S1.** Excluded publication

| Type | Title |
| --- | --- |
| Retracted publication | RETRACTED:Vitamin D Supplementation Affects Serum High-Sensitivity C-Reactive Protein, Insulin Resistance, and Biomarkers of Oxidative Stress in Pregnant Women (Publication with Expression of Concern. See vol. 150, pg. 3042, 2020) (Retracted article. See vol. 151, pg. 1359, 2021) |
|  | RETRACTED:The malignant obesity hypoventilation syndrome (MOHS) (Retracted article. See vol. 15, pg. 358, 2014) |

**Supplementary Table S2.** Top ten authors with the highest TLCS

| Rank | Name | Publications | TLCS | TGCS | Institutions |
| --- | --- | --- | --- | --- | --- |
| 1 | Michael F. Holick | 21 | 1,288 | 3,237 | Boston University |
| 2 | T C Chen | 4 | 1,195 | 2,543 | Boston University |
| 3 | Zhiren Lu | 2 | 1,082 | 2,327 | Boston University |
| 4 | L Y Matsuoka | 1 | 1,042 | 2,258 | Boston University |
| 5 | J Wortsman | 1 | 1,042 | 2,258 | Boston University |
| 6 | Rolf Jorde | 22 | 653 | 2,039 | UiT The Arctic University of Norway |
| 7 | M B Zemel | 17 | 556 | 2,491 | University of Tennessee |
| 8 | Sarah L Booth | 3 | 345 | 772 | Tufts University |
| 9 | Paul Lips | 8 | 338 | 1,520 | Vrije Universiteit Amsterdam |
| 10 | Jack A Yanovski | 6 | 336 | 871 | Tufts University |

**Supplementary Table S3.** Top ten journal with the highest TLCS

| Rank | Journal | Recs | TLCS | TGCS | Impact factor(2022-2023) | H index |
| --- | --- | --- | --- | --- | --- | --- |
| 1 | American Journal of Clinical Nutrition | 70 | 2,436 | 8,948 | 7.1 | 307 |
| 2 | Obesity Surgery | 204 | 2,050 | 7,036 | 2.9 | 128 |
| 3 | Journal of Clinical Endocrinology & Metabolism | 82 | 2,004 | 6,903 | 5.8 | 328 |
| 4 | Obesity | 37 | 814 | 2,153 | 6.9 | 177 |
| 5 | International Journal of Obesity | 52 | 714 | 1,943 | 4.9 | 204 |
| 6 | European Journal of Endocrinology | 20 | 619 | 1,782 | 5.8 | 131 |
| 7 | Journal of Steroid Biochemistry and Molecular Biology | 57 | 507 | 1,512 | 4.1 | 116 |
| 8 | British Journal of Nutrition | 62 | 398 | 2,280 | 3.6 | 166 |
| 9 | Surgery for Obesity and Related Diseases | 46 | 377 | 1,423 | 3.1 | 75 |
| 10 | Journal of Nutrition | 46 | 359 | 2,168 | 4.2 | 240 |
